# Dermatofibrosarcoma protuberans treated by micrographic surgery

**DOI:** 10.1038/sj.bjc.6600643

**Published:** 2002-11-26

**Authors:** A Ah-Weng, J R Marsden, D S A Sanders, R Waters

**Affiliations:** Skin Oncology Service, University Hospital Birmingham NHS Trust, Birmingham B29 6JD, UK

**Keywords:** dermatofibrosarcoma protuberans, micrographic controlled excision, micrographic surgery, recurrence

## Abstract

Dermatofibrosarcoma protuberans is an uncommon cutaneous tumour which rarely metastasises. However, local recurrence following apparently adequate surgical excision is well recognised, presumably as a result of sub-clinical contiguous growth, for which micrographically controlled excision would be a logical treatment. A retrospective study of all patients treated by micrographic surgery, from April 1995–March 2000, at a tertiary skin oncology centre. Twenty-one patients (11 males), age 14 to 71 years with dermatofibrosarcoma protuberans on the trunk (10 patients), groin (four), head and neck (four), and limbs (three) were treated. In 15 patients one micrographic layer cleared the tumour, and four were cleared with two layers. For one patient the second stage was completed by conventional excision guided by positive margins. Another patient with a multiply recurrent perineal dermatofibrosarcoma protuberans, not cleared in one area after two layers, died from a pulmonary embolus before total clearance could be achieved. There was no correlation between tumour size and lateral excision margin. No recurrence was observed during the follow-up, from 21 to 80 months, median 47 months. The study provides further support for micrographic surgery as the treatment of choice for dermatofibrosarcoma protuberans.

*British Journal of Cancer* (2002) **87**, 1386–1389. doi:10.1038/sj.bjc.6600643 www.bjcancer.com

© 2002 Cancer Research UK

## 

Dermatofibrosarcoma protuberans (DFSP) is an uncommon fibrosarcoma which appears to originate from dermal fibroblasts (Taylor and Helwig, 1962; Lautier *et al*, 1990). It grows slowly and contiguously with wide extension between adjacent collagen bundles, and not infrequently into the underlying fascia and muscle. These microscopic infiltrations may not be clinically apparent, and can be missed on histology by using conventional serial vertical sections.

Regional and distant metastases from DFSP do occur but are rare, probably less than 1% (Smola *et al*, 1991), and certainly lower than previous estimates of 4–6% (Roses *et al*, 1986; Rutgers *et al*, 1992). Distant metastases to the lung, even up to 9 years after primary excision, have been described (Taylor and Helwig, 1962). The risk of metastasis appears to increase with repeated incomplete excision and de-differentiation towards a higher-grade fibrosarcoma (Taylor and Helwig, 1962). Fatalities from direct intra-cerebral invasion have also been reported (Mcpeak *et al*, 1967; Barnes and Coleman, 1984).

Treatment of DFSP by local excision consistently results in high recurrence rates of 30–76% (Taylor and Helwig, 1962; Mcpeak *et al*, 1967; Hajdu, 1979; Smola *et al*, 1991; Mark *et al*, 1993). The recommended approach has been wide local excision with lateral margins of at least 3 cm and including the underlying fascia (Mcpeak *et al*, 1967; Bendix-Hansen *et al*, 1983; Roses *et al*, 1986; Rutgers *et al*, 1992). Larger margins do produce better clearance rates but a corresponding greater loss of tissue as there is no reason to believe that the tumour growth is concentric with the clinical margin. A lateral margin of 3 cm results in a local recurrence rate of 10–20% (Mcpeak *et al*, 1967; Roses *et al*, 1986; Gloster *et al*, 1996) and at 4 cm a recurrence rate of 6% was found in 96 patients (Petoin *et al*, 1985). Of 58 cases, even a 10 cm excision width would not have cleared the tumour in two patients (Ratner *et al*, 1997).

The aim of treatment should be complete tumour clearance and maximum preservation of normal tissue. Micrographic controlled excision (MCE) of skin tumours allows examination of all surgical margins, and preliminary evidence for DFSP suggested this method to be effective (Mikhail and Lynn, 1978; Robinson, 1985; Parker and Zitelli, 1995). Results from subsequent series and retrospective studies show a consistently high rate for complete clearance in both primary and recurrent tumours (Hobbs *et al*, 1988; Dawes and Hanke, 1996; Gloster *et al*, 1996; Haycox *et al*, 1997; Ratner *et al*, 1997). Together these reports appear to indicate support for MCE as the most appropriate treatment of DFSP. We present the results of all our patients in support of MCE as the treatment of choice for DFSP.

## MATERIALS AND METHODS

### Clinical data

Diagnosis of DFSP was confirmed histologically and on positive CD34 staining by an experienced dermatohistopathologist (DSAS). Twenty-one patients with DFSP were treated by MCE, undertaken by one operator (JR Marsden), from April 1995 to March 2000. The following characteristics recorded include age at presentation, sex, duration of tumour, clinical size, site and any prior attempt at excision. Also details of inpatient or outpatient care, mode of anaesthesia used, number of micrographic stages, margin and depth of excision, type of repair, complications, cosmesis and functional outcome, were documented. Each subject was recalled for a final outpatient review to corroborate clinical information and update clinical outcome.

### Operative method

The tumour or residual scar tissue was debulked prior to MCE. A modified Mohs micrographic surgical procedure which utilised formalin-fixed, paraffin embedded tissue for an improved interpretation of histology of soft-tissue tumours was employed (Clayton *et al*, 2000). Micrographic layers of 2–3 mm thick were excised to give an initial 1 cm lateral excision margin from the tumour or scar margin, except in one patient where the initial margin was 0.5 cm. The first layer was resected down to the deep fascia. Guided by histologically positive margins, subsequent micrographic layers were excised at 1 cm intervals to minimise the total number of resection stages. These were divided, colour-coded, orientated in tissue cassettes, fixed and embedded in paraffin before staining with haematoxylin and eosin, and for CD34. Sections were reported within 20 h, if necessary further MCE was performed within 24 h until clearance. Analysis for statistical correlation between tumour diameter and lateral excision was undertaken by Minitab statistical package.

## RESULTS

### Clinical characteristics

Of the 21 patients, 11 were male, and the age at presentation 36 (14–71) years, median (range). The duration of onset to presentation was 24 months (6 months to 30 years). Maximum tumour diameter was 3.25 (1.5–16) cm. Clinically, most were typical indurated rubbery pale brown/pink plaques with a smooth surface and often studded with multiple nodules. The sites of presentation were the trunk (10 patients), groin (four), head and neck (four), and limbs (three). The commonest site was upper chest (seven patients).

Most patients were referred with a primary DFSP, however five patients presented with recurrent tumours. Of these three recurred within 18 months of the initial local excision, one had re-grown slowly over the site of an incompletely excised DFSP 20 years previously, and another had a repeatedly recurrent perineal tumour treated unsuccessfully by wide local excision on at least three occasions over 15 years. Eleven were referred from their primary care physician, the majority of whom had not yet had a histological diagnosis. The other patients were sent after a diagnosis had been established on histology. Seven patients had undergone previous resection of the primary tumour which were histologically incompletely excised, and at tertiary consultation showed no visible or palpable tumour.

### Results of micrographic surgery

To achieve the clearance of tumour in 21 patients, 15 required only one micrographic layer, and four needed a second layer. In two other patients, however, total clearance could not be assessed. One had extensive sub-clinical involvement in six out of nine micrographic samples over both lateral and deep margins after the first micrographic layer. Further surgery under local anaesthesia was not possible due to the extent of the lesion and its complex site in the supraclavicular fossa and root of neck. The second excision layer was directed by histologically positive margins, but carried out conventionally. In the other patient a large multiply recurrent perineal DFSP was not cleared in the deep margin after the second micrographic layer. Unfortunately, he died on the 10th post-operative day from a pulmonary embolus before further micrographic surgery could be undertaken.

**Figure 1:**
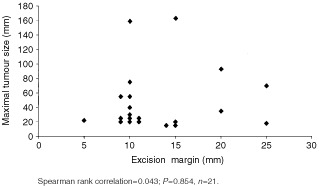
Correlation of maximal tumour diameter to lateral excision margin

Of those cleared after a second layer, two patients required a micrographic layer to both lateral and deep margins, and in the other two further layers to the deep margin only. Further resection to the deep margin involved partial excision of the sternomastoid muscle, full thickness dartos muscle, rectus sheath and full-thickness occipital bone. With the addition of the lateral margin from a prior excision biopsy, measuring 0.5–1.5 cm, recorded in three patients, the total margin of lateral clearance ranged from 0.5 to a maximum of 2.5 cm, median 1.5 cm. This corresponds to a clearance of 0.5 cm in one patient (5%), 1 cm in 12 patients (56%), 1.5 cm for four (19%), 2 cm in one (5%), and 2.5 cm in another (5%). In our cohort, there was no significant statistical correlation evident between maximal tumour size and lateral margin necessary for complete excision (Spearman rank correlation=0.043; *P*=0.854, *n*=21) (Figure 1

). For the first micrographic layer, a median of 12 samples were processed, range four to 76, and in the second layer, a median of six samples, range two to 15.

Sixteen patients were treated under local anaesthesia, five needed regional or general anaesthesia which included the youngest or those with the largest tumours. Twelve were managed as outpatient cases in the day surgical unit, and nine patients required further in-patient care, admission duration 2 (1–10) days, including those who had general anaesthesia.

For 15 patients the surgical defects were closed directly. Four required split skin grafts, one to an overlying pectoralis muscle flap. A patient needed a cavarial graft followed by a scalp rotation flap. Except for the fatality which occurred despite prophylaxis for deep vein thrombosis, the only other early post-operative complication was transient flexion weakness and paraesthesia of the fingers developing distal to the surgical site. At follow-up cosmesis was assessed by the attending physician. In 16 patients the wound cosmesis was described as very good, four as fair compromised by split skin grafts, stretched scars and in another a hypertrophied scar treated satisfactorily with intra-lesional triamcinolone. No permanent functional deficit was noted. There was no recurrence observed during the follow-up interval 47 (21–80) months.

## DISCUSSION

DFSP represents about 1% of all soft tissue sarcomas with an estimated incidence of 0.8/million (Bendix-Hansen *et al*, 1983). A higher standardised incidence rate of 4.5/million from 1970 to 1984, based on crude rates of four female cases was reported in the Rochester population (Tsu *et al*, 1990). In comparison, age standardised incidence data from the West Midlands Cancer Intelligence Unit showed a mean annual rate of 1.8/million for the region between the period 1984 to 1998 (personal communication, Dr Cheryl Hyde) on a crude rate of 142 histologically confirmed cases. Over the corresponding period, we estimate an age standardised incidence of 2.1/million for males and 1.5/million for females, which confirms the previously suggested male predominance in several case series (Pack and Tabah, 1951: Taylor and Helwig, 1962; Mcpeak *et al*, 1967).

The median age at presentation of 36 years is comparable to other reports which observed onset from the third to fifth decade (Taylor and Helwig, 1962; Mcpeak *et al*, 1967). As expected, the time to presentation extends from weeks to decades but was usually several years (Mcpeak *et al*, 1967). The tumour has been described on almost every site on the body surface, typically as an indurated dermal and subcutaneous plaque or nodule. Consistent with previous observations the commonest area in half of all cases was the upper trunk, especially the supra-clavicular fossa (Pack and Tabah, 1951; Taylor and Helwig, 1962; Mcpeak *et al*, 1967, Roses *et al*, 1986; Hajdu, 1979). There are reports describing a history of trauma in 10–20% of cases preceding the development of DFSP (Pack and Tabah, 1951; Taylor and Helwig, 1962). In one of our patients the tumour developed within an old burn scar, and another after a scarring spade injury to his shoulder.

In our study, all the tumours could be completely resected with 2.5 cm lateral margins, but this does not include four out of seven patients who had a prior diagnostic excision biopsy where the lateral margins were not recorded. Other studies of Mohs micrographic excision for DFSP showed a comparable clearance rate for lateral margins (Table 1

**Table 1:**
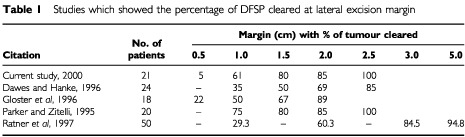
Studies which showed the percentage of DFSP cleared at lateral excision margin

).

Tumour extension into the deep fascia is a common finding and is associated with recurrence. In a series of 20 patients all tumours were cleared by micrographic excision of deep fascia and superficial muscle layer, with those over the forehead and scalp through the periosteum (Parker and Zitelli, 1995). Of 10 patients treated by micrographic surgery, four had invaded muscle of whom two had periosteum involvement (Hobbs *et al*, 1988). In four cases described by Robinson, two had extended into superficial muscle and the others localised in fascia (Robinson, 1985). Excision of fascia were undertaken in six out of 16 (27%) where the tumour recurred, compared to 23 (72%) out of 32 in whom tumour did not recur (Roses *et al*, 1986).

We found no statistical relationship between tumour size and required margin of excision but four patients had prior unrecorded excision biopsy margins. Similarly, in those five patients with recurrent tumours, the size would be underestimated by preceding attempts at clearance. Other studies have not observed a relationship of maximum tumour size to excision margin (Roses *et al*, 1986; Ratner *et al*, 1997). However, one report found tumours less than 2 cm could be safely excised by a 1.5 cm lateral margin (*n*=9), as opposed to those greater than 2 cm which needed 2.5 cm margin (*n*=11) (Parker and Zitelli, 1995). Although in this study eight were recurrent tumours, and the number who had a prior diagnostic biopsy were not specified.

To date no recurrence of DFSP following micrographically controlled excision have been observed after a follow-up interval of 38 (12–71) months in our series. From previous studies of conventional local excision, the majority of recurrences developed early after resection. Approximately half presented within 2 years, and 80% within 3 years (Mcpeak *et al*, 1967). Of 20 patients six recurred at 21 (6–36) months for lateral margins of 1–3 cm (Smola *et al*, 1991). In 98 cases, a 49% recurrence was observed after local resection; 40% within the first year, and 75% in the first 3 years (Taylor and Helwig, 1962). Similar recurrence rates were found by McPeak in 82 patients, and a series of 119 patients treated by wide excision (Mcpeak *et al*, 1967; Hajdu, 1979). The head and neck is a more common site for recurrence, and it is unclear whether this reflects the difficulties in excising DFSP over this area (Barnes and Coleman, 1984; Mark *et al*, 1993). A small number of patients, however, present with a very delayed recurrence, up to 20 years after excision (Mcpeak *et al*, 1967; Mikhail and Lynn, 1978; Roses *et al*, 1986). Of our group, one patient presented with a DFSP on her forehead, at the site of a previously suspected DFSP excised over 20 years earlier.

The current evidence to support MCE for the treatment of DFSP derives from case observations and retrospective studies (Dawes and Hanke, 1996; Haycox *et al*, 1997). The largest study incorporates grouped data of 50 patients from several centres (Ratner *et al*, 1997). Haycox *et al* (1997) reviewed the world-wide literature and found a mean recurrence rate of 2.4% from a total of 169 reported cases. Although most series describe no recurrences the highest reported rate was two out of 24 cases (8.3%) (Dawes and Hanke, 1996), with two other groups each having had one recurrence in 15 (6.6%) and 50 (2%) patients followed-up (Gloster *et al*, 1996; Ratner *et al*, 1997).

Recent retrospective series have revealed promising results for adjuvant radiotherapy in preventing local recurrence of incompletely excised DFSP but its role yet remains to be established (Haas *et al*, 1997; Linder *et al*, 1999).

For day surgery under local anaesthetic, an estimated cost of wide local excision at £460 compared well with one stage of MCE at £560, the only treatment required in 12 patients. Of the nine patients who required overnight stay after MCE the total cost for each was estimated at £1770 per day inclusive of general anaesthesia. The operative time is not any longer for MCE but processing the specimen takes 10–25 min per micrographic layer. Histopathology reporting of micrographic sections is considerably more demanding, from five to 10 times longer than conventional sections, depending on the number of samples. The higher cost of MCE, however, would be outweighed by the improved surgical outcome and prognosis.

In summary, our data provides further support for the favourable outcome following margin control in the excision of DFSP. This approach demonstrates very low recurrence rate with optimum conservation of normal tissue, and would be the treatment of choice for DFSP.

## ACKNOWLEGEMENTS

We are grateful to Dr Cheryl Hyde of the West Midlands Cancer Intelligence Unit, Public Health Building, The University of Birmingham, Birmingham, for providing the incidence data. We would like to acknowledge the help of Mr RA Walsh, consultant surgeon, department of neurosurgery, Queen Elizabeth Hospital, and Mr S Dover, consultant surgeon, department of maxillo-facial surgery, Queen Elizabeth Hospital, for their clinical support.
